# Anatomical variations and accessory structures in the maxilla in relation to implantological procedures: an observational retrospective study of 212 cases using cone-bean computed tomography

**DOI:** 10.1186/s40729-022-00459-7

**Published:** 2022-11-28

**Authors:** Augusto Cimolai-de la Encina, Natalia Martínez-Rodríguez, Ricardo Ortega-Aranegui, Jorge Cortes-Bretón Brinkmann, José María Martínez-González, Cristina Barona-Dorado

**Affiliations:** 1 Department of Conservative Dentistry and Orofacial Prosthodontics, Faculty of Dentistry, Complutense University of Madrid, Madrid, Spain; 2 Department of Dental Clinical Specialties, Faculty of Dentistry, Universidad Complutense de Madrid, Pza Ramon y Cajal S/N, 28040 Madrid, Spain; 3 Surgical and Implant Therapies in the Oral Cavity Research Group; University Complutense, Madrid, Spain

**Keywords:** Cone-beam computed tomography, Maxillary sinus septa, Canalis sinuosus, Posterior superior alveolar artery, Maxillary sinus lift

## Abstract

**Purpose:**

This study used cone-beam computed tomography (CBCT) to analyze the prevalence of several maxillary anatomical/accessory structures, as well as variations within each type, assessing how accurate diagnosis can minimize the risk of intraoperative complications during implantological procedures in the oral cavity.

**Methods:**

212 CBCT scans of the maxilla were analyzed, captured over a period of 18 months for surgical planning purposes. The prevalence of posterior superior alveolar arteries (PSAA), maxillary sinus septa (MSS), and branches of the canalis sinuosus (CS) were evaluated, as were the diameter and location of each anatomical structure in horizontal and vertical planes. *P* < 0.05 was considered statistically significant.

**Results:**

PSAAs were observed in 99.1% of cases, the intrasinus type being the most frequent; MSS were noted in 15.6% of the sample, mainly in the posterior region with sagittal orientation; CS branches were observed in 50% of patients, mainly in relation to the incisors and significantly more prevalent among males.

**Conclusions:**

The use of CBCT significantly increases the possibility of clearly identifying these anatomical structures. The differences found between patients highlight the importance of carrying out an exhaustive radiological study of the individual to prevent complications, such as Schneiderian membrane perforation, neurovascular damage or bleeding during surgery.

**Graphical Abstract:**

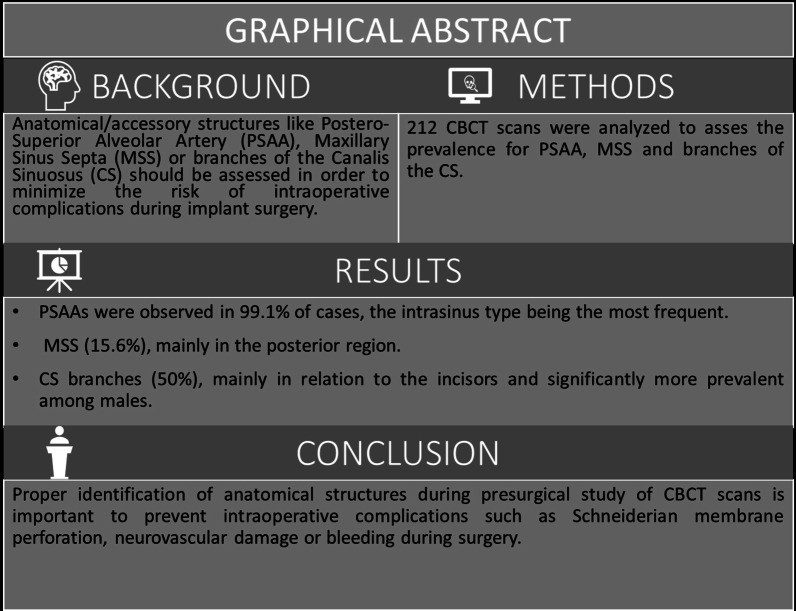

## Background

During any surgical procedure, the secondary objective is always to prevent adverse events, minimize damage, and improve the overall outcome for the patient [1, 2]. Adverse events reported in the course of dental treatment are mainly due to surgical procedures including oral surgery and implantology; these occur in some 50–60% of cases. Furthermore, according to the Spanish Observatory for Patient Safety in Dentistry (SOPSD), 40% of these adverse events are the result of errors in diagnostic planning or poor technical execution [3–6].

In the maxillary region, tooth loss leads to maxillary sinus pneumatization, especially in cases of severe atrophy. Often, this pneumatization can be dealt with by means of bone regeneration techniques, e.g., maxillary sinus floor augmentation (MSFA) with a lateral approach before rehabilitating the area with osseointegrated implants [7].

This region is known to present considerable anatomical variation with regard to the posterior superior alveolar artery, a branch of the maxillary artery, and its relationship with the lateral wall of the maxillary sinus. The latter is classified as three types: Type I (intrasinus); Type II (intraosseous); and Type III (superficial) [8].

Moreover, the presence of septa or bony partitions inside the maxillary sinus create regions of Schneiderian membrane adhesion and irregularities, which can make MSFA procedures difficult, leading to a higher incidence of intra-operative complications [9].

**Fig. 1:**
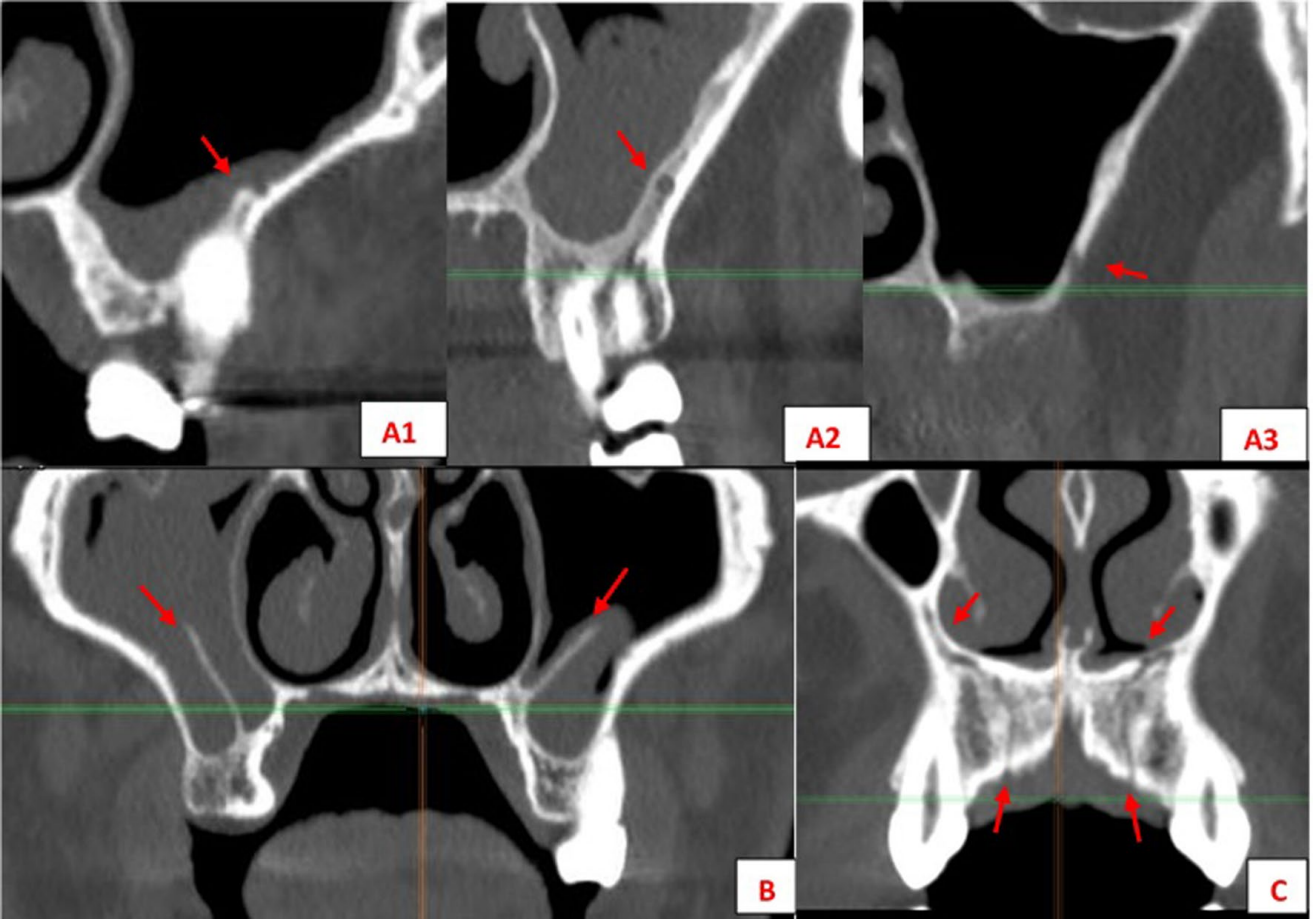
Anatomical structures evaluated: PSAA variations (**A**) 1–2–3; (**B**) MSS, (**C**) CS

**Fig. 2:**
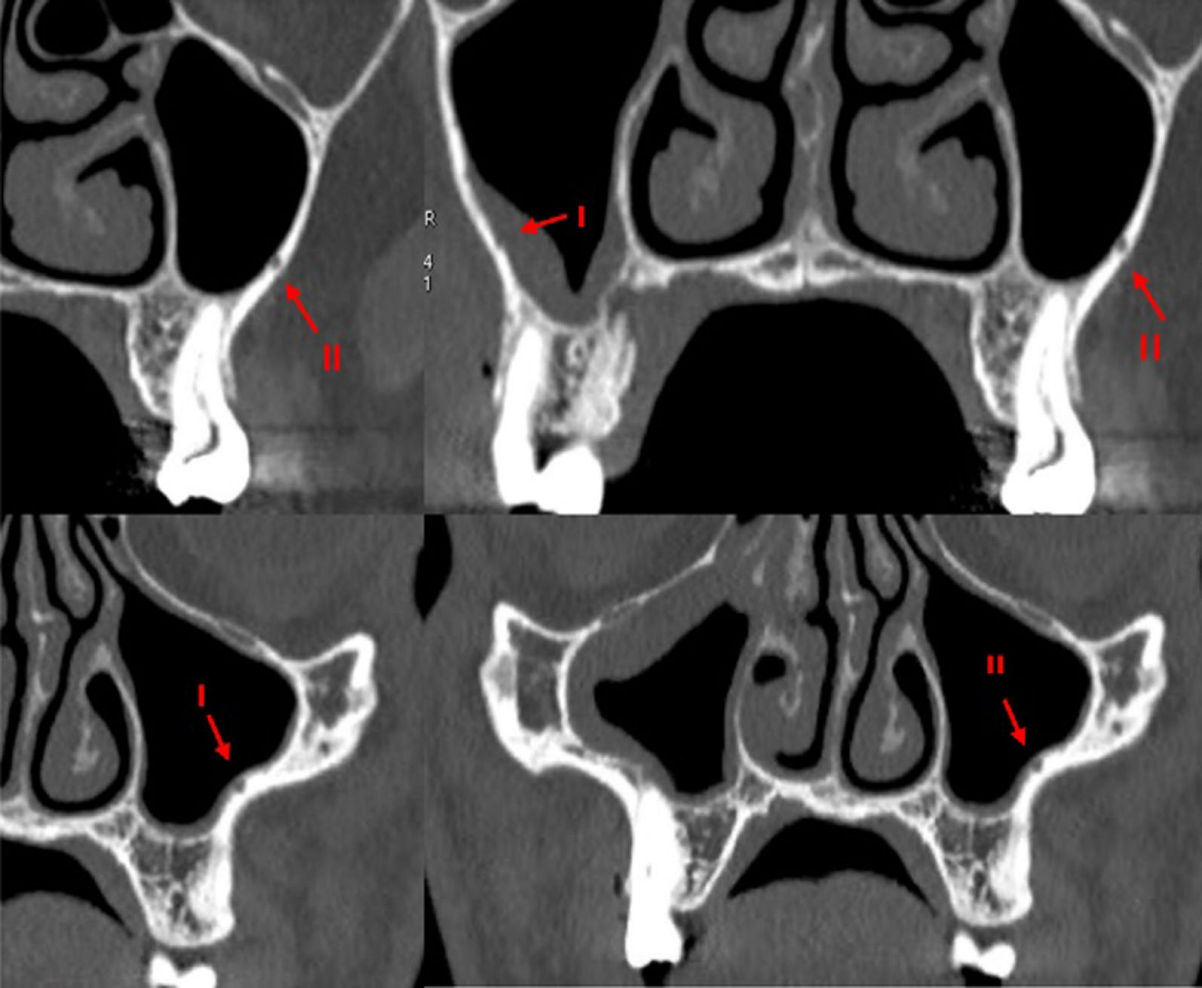
Different variations of PSAA (I/Intrasinus; II/Intraosseous) seen in sagittal planes

**Fig. 3:**
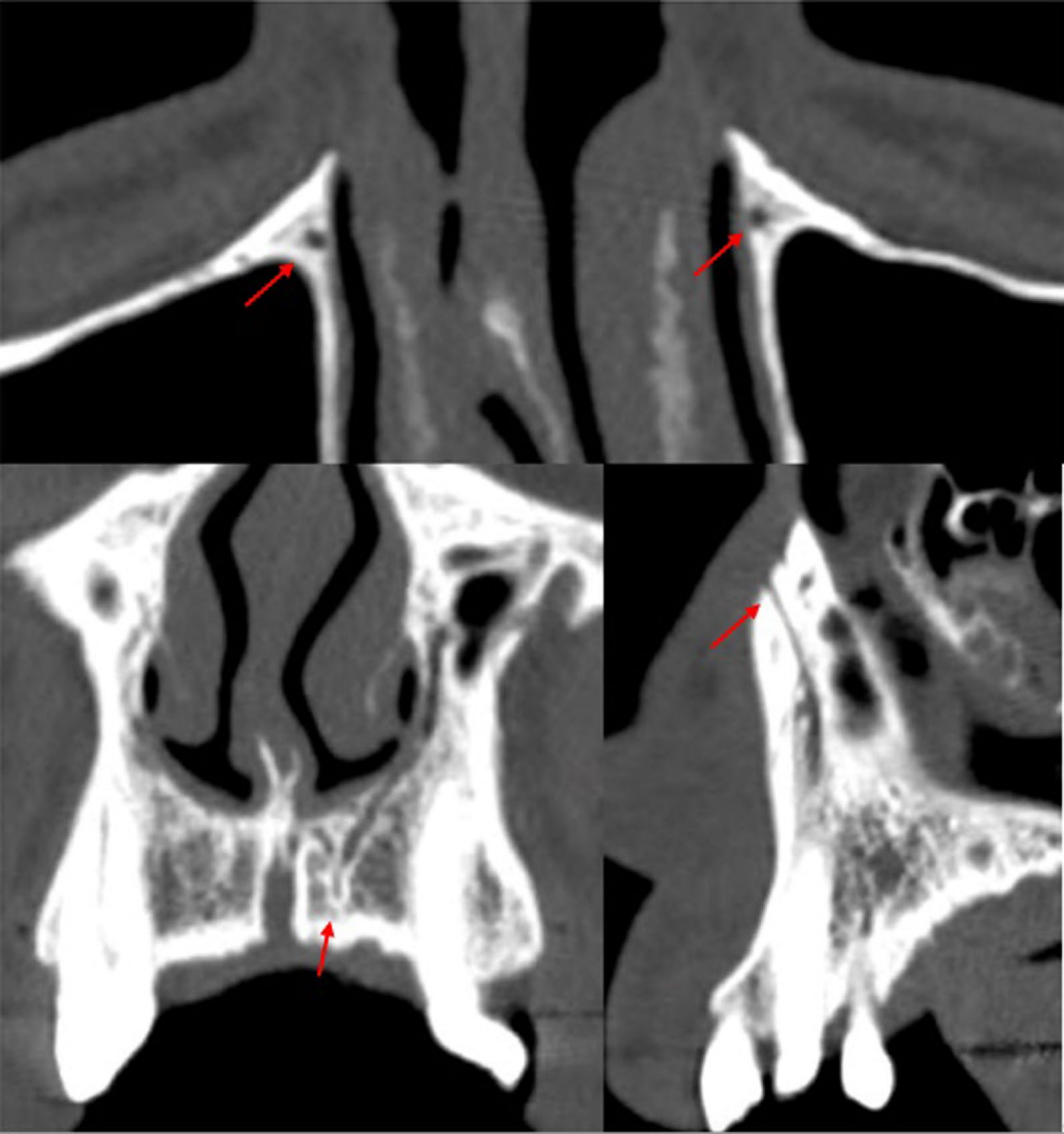
CS branches course from the infraorbitary canal to the hard palate in the maxilla seen in different planes (axial, frontal, sagittal)

Another anatomical feature exhibiting considerable variation is the canalis sinuosus, located in the anterior maxillary region (premaxilla) (Figs. 1, 2, 3). It originates in the infraorbital canal and runs caudally and medially to the nasopalatine canal, occasionally presenting branches of the anterior superior alveolar neurovascular bundle along its entire path through the premaxilla [10, 11].

Unless these anatomical variations in the maxilla are correctly diagnosed in advance, they can lead to a higher incidence of complications, including bleeding, nervous and traumatic problems, and the subsequent failure of implant and regenerative procedures. In this context, radiographic diagnosis and planning will play a fundamental role in minimizing the risk of intraoperative complications during surgical procedures. CBCT provides a reliable means of making a preliminary study to identify those structures that are undetectable with two-dimensional imaging techniques. It will also eliminate overlaps and artifacts and so facilitate a more comprehensive diagnosis and treatment plan [12–16].

In this context, the primary outcome of the present study was to evaluate the prevalence of different anatomical variations and accessory structures in the maxilla using CBCT, being the secondary outcome to determine and evaluate to what extent this accurate diagnosis can minimize the risk of intraoperative complications during implant procedures. Consequently, the null hypothesis would be to expect no major differences in the prevalence of these anatomical structures diagnosed with CBCT compared to other radiographic techniques.

## Materials and methods

### Study design

This observational, cross-sectional, retrospective study investigated a patient sample referred by the Radiology Department at the Faculty of Dentistry, Complutense University of Madrid (Spain), selecting CBCT scans taken for preoperative assessment before implant placement, or regenerative procedures, such as MSFA. The study protocol was approved by the Ethics Committee at the San Carlos Hospital, Madrid (C.I. 21/497-E). Informed consent was obtained from all patients whose CBCTs were used.

A total of 212 CBCT studies of the maxilla were collected over a period of 18 months. The sample included 95 men and 117 women.

Any CBCT scans of patients with severe alveolar bone resorption, a history of maxillofacial trauma or surgery, craniofacial syndromes or pediatric patients were excluded, as were scans with insufficient image quality or containing numerous artifacts.

### Radiology equipment and evaluation software

A Cone-Beam 3-D Dental Icat Next Generation® (*Imaging Sciences International, Inc., Hatfield, Pennsylvania, USA*) CBCT unit was used to capture the scans using the following standardized parameters:

- Voltage: 100 kV; current: 5 mA.
- Sensor: flat, amorphous silicon detector panel with a CsI scintillator, 20 × 25 cm.
- Field of view (FOV): cephalometric, 17 × 23 cm.
- Voxel size: 0.2–2.4 mm.
- Reconstruction method: cylindrical.

CBCT imaging data were stored in DICOM format and interpreted using Ez3D Plus® software (Vatech & Ewoo, Gyeonggi-do, South Korea).

### Measurement method

All scans were assessed in three different display formats:

- Multiplanar reformation (MPR) displays the entire tomographic volume in axial, sagittal and coronal planes.
- Panoramic reconstruction, with three slice thicknesses (3, 5, and 10 mm).
- Three-dimensional reconstruction.

**Fig. 4:**
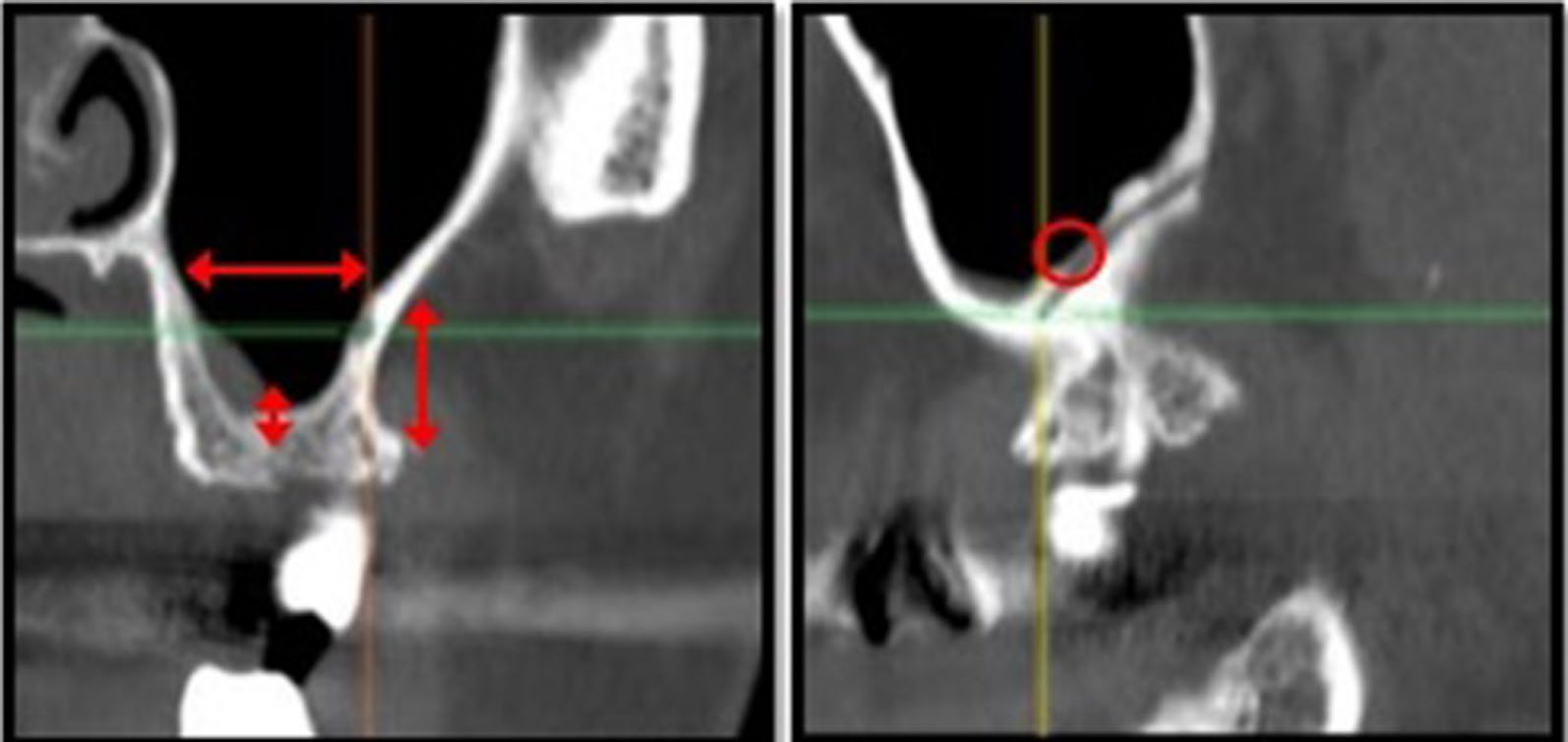
Viewing and measuring the PSAA in MPR format (axial and sagittal planes). Horizontal and vertical measures related to PSAA

**Fig. 5:**
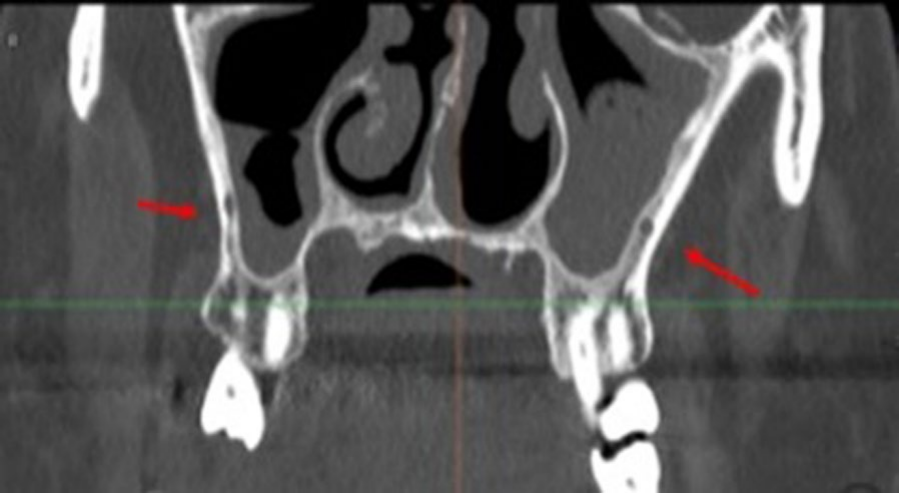
Left: Intrasinus (I) PSAA. Right: intraosseous (II) PSAA

**Fig. 6:**
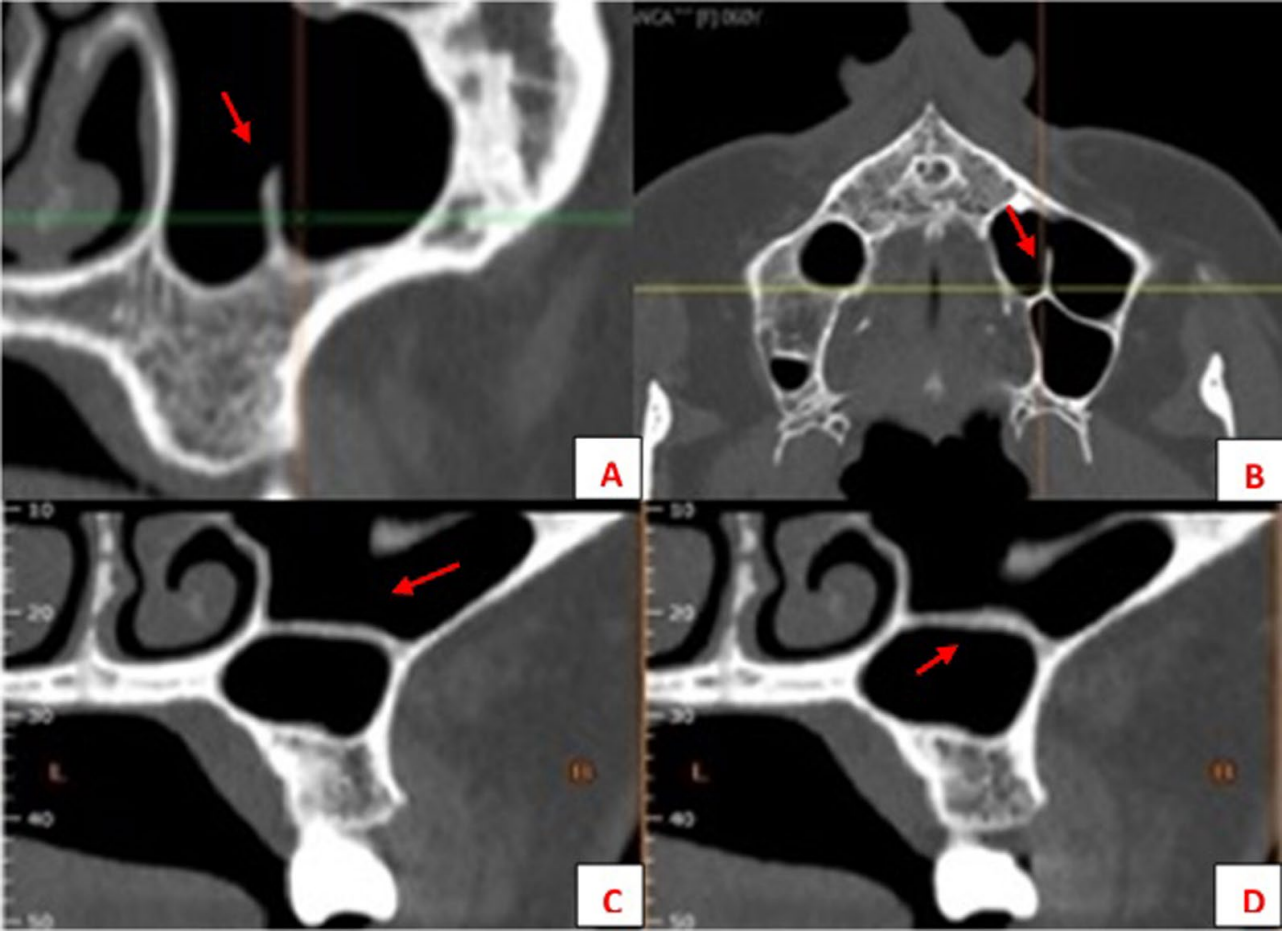
Different orientations of MSS: (**A**) sagittal, (**B**) sagittal + bucopalatal, **C**, **D** transversal

**Fig. 7:**
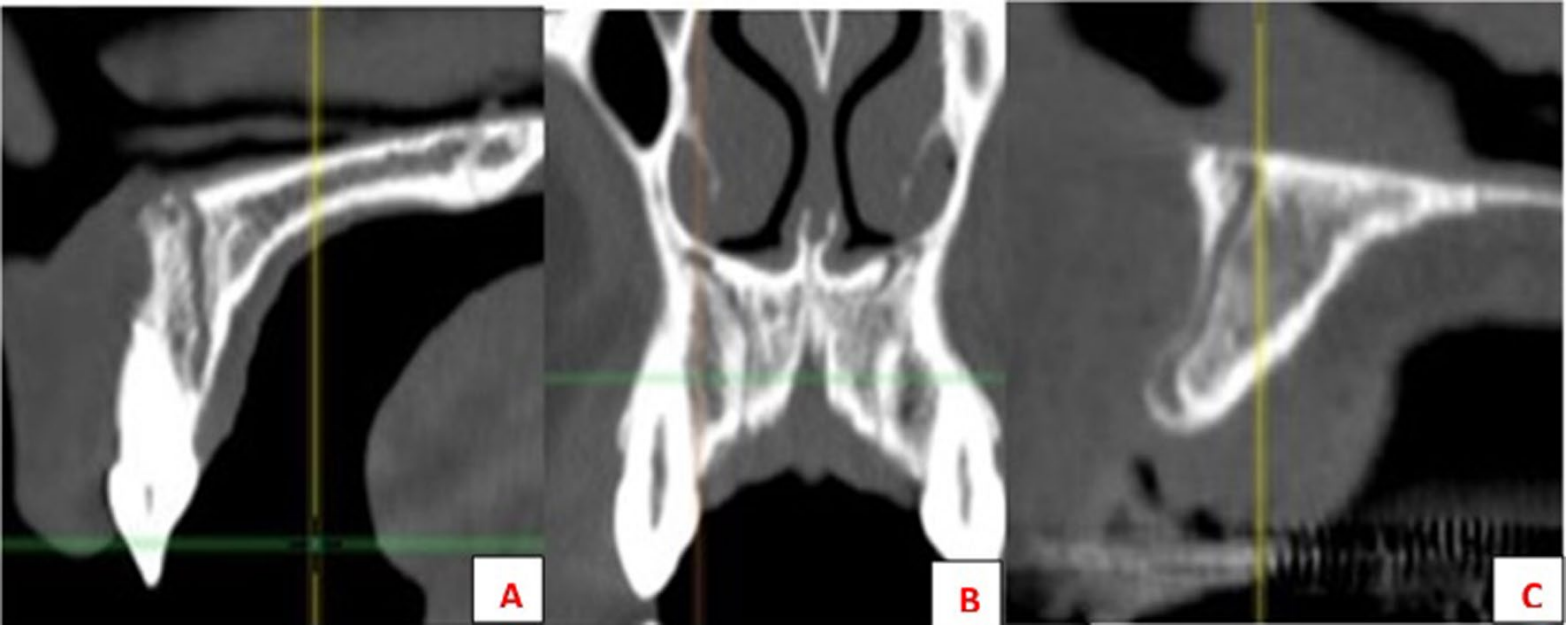
CS branches at different locations: Parallel to central incisor (**A**); Canine area bilaterally (**B**); Alveolar crest (**C**)

The only demographic variable recorded was patient gender. Specific analyses were carried out for each of the following variables:

- Posterior superior alveolar artery, studying the relationship between the artery and lateral wall of the maxillary sinus observed in the most frequent area to perform a MSFA procedure, upper molar area or middle to posterior region of the maxillary sinus, (intrasinus/Type I, intraosseous/Type II, and superficial/Type III), the artery’s diameter and horizontal distance (in mm) to the medial wall of the maxillary sinus, and vertical separation from the alveolar ridge. Measurements were taken with tools included in the software (Figs. 4, 5).
- Maxillary sinus septa, recording prevalence, location (anterior, middle or posterior) and orientation (sagittal, buccopalatal, or transversal) within the maxillary sinus, as well as their association with the presence of teeth (Fig. 6).
- Branches of the canalis sinuosus (CS): studying prevalence, diameter (mm) and its relationship with tooth emergence (Fig. 7).

### Statistical analysis

Statistical analysis was performed with SPSS software for Windows (IBM®, USA). Mean values and standard deviations were recorded for each variable. The Chi-squared test was applied to analyze differences in relation to gender. The Kappa coefficient was used to evaluate concordance between the three display formats that were visualized by three experienced clinicians (A.C.E., R.O.A., J.M.M.G).

Student’s *t* test and one-way ANOVA were applied to assess specific characteristics of the posterior superior alveolar artery. The study sample presented a statistical power of 80% with a confidence level of 95%. A *p* value of less than 0.05 was considered statistically significant.

## Results

### Posterior superior alveolar artery

PSAAs were observed in both hemimaxillae in 99.1% of the sample (210 scans), with a prevalence by gender of 98.3% in females and 100% in males. The type of PSAA found most frequently in both hemimaxillae was Type I (intrasinus), followed by Type II (intraosseous) (Fig. 5).

The horizontal and vertical measurements of the artery’s location with respect to other anatomical structures are shown in Table 1.

**Table 1:** Descriptive results for PSAA

Gender	Male	95 (100%)	
	Female	115 (98.3%)	
Total		210 (99.1%)	
		Right	Left
Type	Intrasinus (I)	120 (57.1%)	104 (49.5%)
	Intraosseous (II)	75 (35.7%)	80 (38.1%)
	Superficial (III)	15 (7.1%)	26 (12.4%)
Diameter	Mean, mm ± SD	1.1 ± 0.4	1.1 ± 0.4
	< 1 mm	49%	47.6%
	1–2 mm	47.1%	49.5%
	> 2 mm	3.8%	2.9%
Distance to medial wall, mm ± SD		14.1 ± 7	15.3 ± 1.4
Distance to alveolar ridge, mm ± SD		15.9 ± 10.9	16.5 ± 1.4
Residual alveolar ridge, mm ± SD		8.6 ± 3.2	8.5 ± 3.2

Inferential statistics applied to the horizontal and vertical measurements of the PSAA did not find any association between the variables. In fact, they were found to be totally independent of one another.

The Kappa concordance coefficient returned null results (0). While the initial detection of the PSAA was practically absolute (99.1%) using the MPR format, both the panoramic and 3D reconstruction formats were effectively unable to detect the artery (0.5% and 0%, respectively).

### Maxillary sinus septa

Maxillary sinus septa were present in 15.6% of the sample (33/212 CBCT studies), with no statistically significant differences between genders. Of these, 39.4% were situated on the right, 24.2% on the left, and 36.2% were bilateral.

In terms of location, they were most commonly found in the posterior region of the sinus with a sagittal/vertical orientation and mainly with primary origin or secondary to tooth loss (Table 2).

**Table 2:** Descriptive results for MSS

Gender	Male	14 (14.7%)	
	Female	19 (15.6%)	
Total		33 (15.6%)	
		Right	Left
Location	Anterior	5 (20%)	3 (15%)
	Middle	8 (32%)	5 (25%)
	Posterior	12 (48%)	12 (60%)
Orientation	Buccopalatal	9 (36%)	4 (20%)
	Horizontal	5 (20%)	5 (25%)
	Sagittal	11 (44%)	11 (55%)
Origin	Secondary	9 (36%)	6 (30%)
	Primary	16 (64%)	14 (70%)

The Kappa concordance coefficient between MPR and panoramic reconstruction formats at different slice thicknesses ranged from 0.6 to 0.8, while this was 0.05 for both MPR and 3D reconstruction formats.

### Branches of the canalis sinuosus

Canalis sinuosus branches were observed in 50% of the sample (106/212 CBCT studies). There were significant differences between the genders, with greater prevalence among men.

Most cases had branches in both maxillae (58.5%), mainly emerging in the palatal region, with a diameter that was typically less than 1 mm (0.9 ± 0.4). Over 40% of septa were related to the central incisor on both sides (Table 3).

**Table 3:** Descriptive results for the CS branches

Gender		
Male	57 (53.77%)	
Female	49 (46.23%)	
Total	106 (50%)	
Side		
Right	25 (23.6%)	
Left	19 (17.9%)	
Bilateral	62 (58.5%)	
	Right	Left
No ref	3 (3.5%)	2 (2.6%)
CI	35 (41.2%)	37 (47.4%)
LI	25 (29.4%)	20 (25.6%)
CA	14 (16.5%)	12 (15.4%)
1PM	7 (8.2%)	6 (7.7%)
2PM	1 (1.2%)	1 (1.3%)
Diameter, mm ± S	0.9 ± 0.4	

*CI* incisor, *CA* canine, *1PM* first premolar, *2PM* second premolar

The Kappa concordance coefficient between the MPR and panoramic and 3D reconstruction formats ranged from 0 to 0.2.

## Discussion

This study aimed first to evaluate the prevalence of different anatomical variations and accessory structures in the maxilla using CBCT and secondly to assess the extent to which accurate diagnosis can minimize the risk of intraoperative complications during implantological procedures in the oral cavity.

Regarding PSAA variations in relation to MSFA augmentation procedures, it should be noted that excessive bleeding is an adverse event that can interfere with the efficiency of the intervention, whether due to reduced visibility, compromised blood supply, graft viability, or by increasing the likelihood of sinus membrane perforation [17, 18]. Therefore, knowing the precise location of the PSAA prior to surgery is essential to minimizing the risk of such complications. In the present study, PSAA was observed in 99.1% of cases.

This high prevalence could be justified because CBCT with adequate resolution allows locating accessory structures in a proportion hitherto unimaginable with other radiological techniques. This fact will acquire even a greater importance in cases where a meticulous and detailed search is carried out, studying the structures simultaneously in multiplanar images and using different filters and tools.

Our results concurs with findings published by Anamali et al. [19] and Ilgüy et al. [20] who detected the PSSA in 85–100% of cases. There is a substantial improvement in detection when CBCT is used in comparison with conventional computed tomography (CT), which only detects 55–64.5% of cases [21, 22]. With respect to the type of PSSA observed in our study, Type I (intrasinus) was the most commonly reported (around 53%), while Type II (intraosseous) was observed in approximately 37% of the sample and Type III (superficial) in only 10%. These results agree with those obtained by other authors who found that intrasinus PSAA was the most common type, observed in some 50% of cases [23–25]. However, Varela Centelles et al. [17] and Rosano et al. [26] observed a higher prevalence of the intraosseous type, reaching values of 100%. Nevertheless, all authors agree with the fact that Type III presents the lowest prevalence [17, 22–24].

Regarding the clinical implications and the risk of copious bleeding during MSFA with lateral approach, Type I represents a greater risk when detaching the membrane, while Type II poses a risk when following the window osteotomy protocol [23–25]. Type III is associated with a risk of bleeding during incision and elevation of the flap [17, 18, 22–24]. Therefore, knowing the precise location of PSAAs enables the surgeon to adopt a more cautious approach at relevant stages of the surgical procedure.

Knowing the exact size (diameter) of the PSSA will be clinically helpful, given that various authors have reported that arteries with a diameter of over 1 mm increase the risk of bleeding by up to 57%, a value which is even higher when the diameter exceeds 2 mm [21, 27–29]. Our results are consistent with those of other authors who found arteries in excess of 1 mm in diameter in over half of cases assessed using CBCT in patient samples similar to the present work [24, 30, 31]. Knowing the precise height of the artery above the alveolar ridge is also clinically relevant as this dictates the limits of the sinus access window. In the molar region, which usually represents the artery’s lowest point and coincides with the access window area, a height of 15 mm has been suggested as a safe limit to avoid damaging the artery during osteotomy [32–34]. Although the values in the present study were above the 15 mm safety limit, this will obviously depend on the extent of the residual alveolar ridge. In many clinical scenarios the values fall below the safety limit (and are closer to 10 mm), so represent a higher risk of vascular damage [35].

The present study did not observe any association between the various PSAA measurements analyzed. The independence of these variables suggests that no correlations can be established between the specific types of PSAA and patient gender, artery diameter, or residual alveolar ridge characteristics. These findings are in line with those of similar studies [30, 36]. At the same time, the enormous variation in PSAA data, together with the fact that other authors do not always agree with our data (Table 4), highlight the importance of correct diagnosis in terms of the artery’s position and features. This will help to avoid possible vascular damage that would have to be resolved by applying gauze or cauterizing the bleeding artery, and could have adverse postoperative consequences [37, 38].

**Table 4:** Review of PSAA data from other studies

Study	Sample	No. maxillary sinuses	% detection		Most frequent type	Ø (mm)		Ridge (mm)
Present study	212 CBCT	424	99.10%	I		1.1	16.2	
Anamali et al. [19]	254 CBCT	508	90.90%	No data		No data	No data	
Elian et al. [32]	50 CT	100	52.90%	II		No data	16.4	
Güncü et al. [22]	121 CBCT	242	64.50%	II		1.3	18	
Ilgüy et al. [20]	135 CBCT	270	89.30%	II		0.94	16.88	
Kang et al. [33]	150 CT	150	64.3			1.18	17.03	
Khojastepour et al. [23]	150 CBCT	211	70.30%	I		0.98–1.52	15.72–17.25	
Lozano-Carrascal et al. [25]	284 CBCT	568	48.60%	I		< 1 mm	13.15	
Mardinger et al. [21]	104 CT	208	55%	I		< 1 mm	19.59	
Rosano et al. [26]	15 CT, cadavers	30	100%	II		< 1 mm	11.25	
Şimşek Kaya et al. [24]	114 CBCT	228	87.70%	II		1–2 mm	15.6	
Varela-Centelles et al. [30]	120 CBCT	240	100%	II		1.3	No data	

Regarding MSS, the main purpose of determining their location is to avoid iatrogenic perforation of the Schneiderian membrane during MSFA with lateral approach [39, 40]. Sinus septa may also hinder the withdrawal of the bone cover from the lateral window to gain access to the maxillary sinus [41].

During MSFA with lateral approach, perforations of the sinus membrane may occur due either to iatrogenic causes derived from incorrect surgical handling or to anatomical considerations inherent to the individual patient, such as reduced thickness, reduced friability, the elasticity of the membrane or the presence of these sinus septa [42, 43]. Regarding the latter, Zijderveld et al. [44] reported five perforations associated with the presence of septa in a total of 11 membrane perforations resulting from 100 sinus lifts. Similarly, several authors conducting retrospective studies have observed a significant association between the presence of sinus septa and membrane perforations [39, 45].

The prevalence of septa in the literature varies widely depending on the radiographic technique used from 21.6% to 68.4% [46–50]. However, in the present study, maxillary sinus septa were present in only 15.6% of the sample. These differences may be attributed to the arbitrary nature of the threshold established to differentiate between irregularities in the maxillary sinus floor and an actual septum of sufficient size and projection within the maxillary sinus.

As far as the location of the septa is concerned our findings agree with most authors (Table 5), who generally observe that most septa are located posteriorly or at the level of the first or second molar. We also found that the predominant orientation of septa was in the sagittal plane, as did Rosano et al. [51]. The second most common orientation in our sample was buccopalatal; this orientation has been described as the most prevalent inclination in other CBCT studies and horizontal septa were the least frequent in all the studies reviewed [24, 50, 52–57].

**Table 5:** Review of MSS data from other studies

Study	Sample	No. maxillary sinuses	% septa	Most frequent type	Location	Origin
Present study	212 CBCT	424	15.60%	Sagittal	Posterior	Primary
Bornstein et al. [52]	212 CBCT	294	66.50%	BP	Middle	No data
Ella et al. [48]	40 + 35 CT, cadavers	150	39%	No data	No data	No data
Hong et al. [34]	139 CBCT	224	38.30%	BP	Anterior	Primary
Hungerbühler et al. [53]	301 CBCT	602	38.90%	BP	Middle	Secondary
Irinakis et al. [45]	79 CBCT	158	48.10%	BP	Middle	Secondary
Krenmair et al. [56]	265 CT	265 CT	27.70%	No data	No data	Secondary
Park et al. [54]	200 CT	400	37%	BP	Middle	No data
Qian et al. [55]	506 CBCT	1,012	48%	BP	Middle	No data
Rosano et al. [51]	30 cadavers	60	39%	Sagittal	Anterior	No data
Schriber et al. [49]	50 CBCT	100	50%	BP	Middle	Primary
Shibli et al. [46]	1,024 OPG	2,048	21.58%	No data	No data	Secondary
Sigaroudi et al. [50]	222 CBCT	444	68.40%	BP	Middle	No data
Talo Yldirim et al. [47]	1,000 CBCT	1,000	29.70%	BP	Middle	Primary

However, it seems evident that carrying out a previous study to precisely recognize sinus septum is essential to minimize intraoperative complications. In this way the surgical approach could vary and change the design of the access window or even recommend the realization of two windows, proximal and distal to the septum. Authors such as Manderalis et al. [58] and Goodacre et al. [59] have developed surgical guides in order to minimize these perforations. Nevertheless, transferring the exact position of the septum from CBCT to the clinical presentation is still challenging. Hence, Texeira et al. [60] recently described an improved computer-guided sinus approach based on a magnetic stackable surgical guide (SSG) aimed at enhancing the safety and efficacy of these procedures. This technique allows access to extensive grafting areas. Thus, a minimally invasive approach using piezoelectric surgery associated with a three dimensionally (3D) printed SSG is used to precisely locate the sinus septum and optimally position the lateral windows.

Finally, the main reason for detecting branches of the CS is to avoid neurovascular injuries during implant placement, regenerative procedures, impacted tooth extractions, or periodontal or periapical surgery in the anterior maxillary region [61, 62]. In the present patient sample, these accessory structures were diagnosed in over half of all cases. Similarly, in a sample of 1000 CBCT scans, Machado et al. [63] reported a prevalence of 52.1%, while Aoki et al. [64] detected an even higher percentage, 66.5% of cases in a sample of 200 CBCT scans. These authors also observed a higher prevalence among men, as in the present work. Other CBCT-based studies support this finding, reporting higher rates of this variation among men than women (15.7–34.7%) [65, 66]. Disparities in image quality and capture as well as the voxel size of the CBCT scanners used may explain the wide range of prevalence cited in the literature [67].

Regarding the diameter, bilateral appearance, and position of branches of the canalis sinuosus, the present results were consistent with most published studies. The branches were generally found to run parallel to the nasopalatine canal, close to the upper central incisors, with a diameter of less than 1 mm, and were mainly observed to be bilateral (Table 6) [61, 64, 65].

**Table 6:** Review of data on CS branches from other studies

Study	Sample	% with branches	% bilateral	Distribution	Diameter (mm)	Difference between Genders
Present study	212 CBCT	50	58.50%	CI > LI > CA > …	0.9	♂
Aoki et al. [64]	200 CBCT	66.5	54.14	CI > LI > CA > …	< 1	♂
Gurler et al. [61]	111 CBCT	100	100	LI > CI > CA	1.37	♂
Machado et al. [63]	1,000 CBCT	52.1		CI > LI > CA > …	1.19	No
Manhâes Junior et al. [72]	500 CBCT	36.2		Midline		No
De Oliveira-Santos et al. [10]	178 CBCT	15.7	21%	CI > CA	1.4	No
Orhan et al. [62]	1,460 CBCT	70.8		CA > ML > CI		
TomrukÇu et al. [66]	326 CBCT	34.7		LI > CI > CA…	1.3	♂
Von Arx et al. [65]	176 CBCT	7.8	56.70%		1.31	No

It should be noted that due to their characteristics, it is difficult to assess canalis sinuosus branches using 2D, periapical or panoramic X-rays. This makes tomography a necessity. It also explains the low levels of concordance obtained using pseudo-panoramic reconstructions and why these structures are easily overlooked [68–72].

## Conclusions

Within the limitations of the present study, it may be affirmed that the use of CBCT significantly increases the possibility of identifying anatomical variations and relationships in the maxilla, minimizing the risk of intraoperative complications during implantological procedures. For this reason, it is very important to carry out an exhaustive radiological study of the individual patient. Intrasinus arteries are the most prevalent type of PSAA. Maxillary sinus septa are mainly located in the posterior sector with a sagittal/vertical orientation. More than half of all patients present branches of the canalis sinuosus, which are more prevalent in men than women and are located mainly at the level of the incisors.

## Data Availability

All data are available in the manuscript and additional files.

## Abbreviations

CBCT: Cone-beam computed tomography
CS: Canalis sinuosus
CT: Computed tomography
FOV: Field of view
MPR: Multiplanar reformation
MSFA: Maxillary sinus floor augmentation
MSS: Maxillary sinus septa
PSAA: Posterior superior alveolar artery
SOPSD: Spanish Observatory for Patient Safety in Dentistry
